# A Digital Modality Decision Program for Patients With Advanced Chronic Kidney Disease

**DOI:** 10.2196/12528

**Published:** 2019-02-06

**Authors:** Ruth Dubin, Anna Rubinsky

**Affiliations:** 1 San Francisco Veterans Affairs Medical Center San Francisco, CA United States; 2 University California San Francisco San Francisco, CA United States; 3 Kidney Health Research Collaborative University of California San Francisco / San Francisco Veterans Affairs Medical Center San Francisco, CA United States

**Keywords:** chronic kidney disease, end-stage renal disease, online social networking, patient education, renal dialysis

## Abstract

**Background:**

Patient education regarding end-stage renal disease (ESRD) has the potential to reduce adverse outcomes and increase the use of in-home renal replacement therapies.

**Objective:**

This study aimed to investigate whether an online, easily scalable education program can improve patient knowledge and facilitate decision making regarding renal replacement therapy options.

**Methods:**

We developed a 4-week online, digital educational program that included written information, short videos, and social networking features. Topics included kidney transplant, conservative management, peritoneal dialysis, in-home hemodialysis, and in-center hemodialysis. We recruited patients with advanced chronic kidney disease (stage IV and V) to enroll in the online program, and we evaluated the feasibility and potential impact of the digital program by conducting pre- and postintervention surveys in areas of knowledge, self-efficacy, and choice of ESRD care.

**Results:**

Of the 98 individuals found to be eligible for the study, 28 enrolled and signed the consent form and 25 completed the study. The average age of participants was 65 (SD 15) years, and the average estimated glomerular filtration rate was 21 (SD 6) ml/min/1.73 m^2^. Before the intervention, 32% of patients (8/25) were unable to make an ESRD treatment choice; after the intervention, all 25 participants made a choice. The proportion of persons who selected kidney transplant as the first choice increased from 48% (12/25) at intake to 84% (21/25) after program completion (*P*=.01). Among modality options, peritoneal dialysis increased as the first choice for 4/25 (16%) patients at intake to 13/25 (52%) after program completion (*P*=.004). We also observed significant increases in knowledge score (from 65 [SD 56] to 83 [SD 14]; *P*<.001) and self-efficacy score (from 3.7 [SD 0.7] to 4.3 [SD 0.5]; *P*<.001).

**Conclusions:**

Implementation of a digital ESRD education program is feasible and may facilitate patients’ decisions about renal replacement therapies. Larger studies are necessary to understand whether the program affects clinical outcomes.

**Trial Registration:**

ClinicalTrials.gov NCT02976220; https://clinicaltrials.gov/ct2/show/NCT02976220

## Introduction

An important part of care for patients with moderate to advanced chronic kidney disease (CKD) is education about treatment options for end-stage renal disease (ESRD) including in-center or in-home renal replacement therapies as well as nondialysis treatment options including kidney transplant or conservative management with no dialysis. Unfortunately, rather than planning ahead for ESRD, up to 50% of patients have emergent starts or undergo unplanned, urgent starts to dialysis [1,2]. Circumstances associated with such unplanned or crash initiation of dialysis, such as hyperkalemia, and the higher infection rates associated with hemodialysis catheters place patients at high risk for frequent hospitalizations and death [3] and impose a high cost on the medical system [1,4]. Furthermore, of the 500,000 patients who have ESRD and receive dialysis, less than 10% utilize in-home dialysis therapies such as peritoneal dialysis or home hemodialysis [5]. Surveys of patients with ESRD on hemodialysis suggest that low patient awareness may be a barrier to choosing self-care in-home dialysis (such as peritoneal or home hemodialysis) compared with in-center dialysis [6].

Studies have shown that predialysis education may increase the likelihood of patients undergoing planned, elective dialysis starts [7]. Predialysis educational programs have been shown to increase the likelihood that patients choose self-care dialysis or in-home dialysis rather than in-center dialysis [8], and they have been shown to increase the likelihood of patients choosing peritoneal dialysis rather than hemodialysis [9-11]. Successful programs have included one-on-one contact with a clinician educator [10]; patient group sessions to allow for discussion [8]; and multidisciplinary education involving an integrated team of doctors, nurses, and social workers [12]. These approaches to dialysis education are effective but may not be accessible to a broad spectrum of CKD patients because of cost and geographic restrictions. Digital technology has the potential to integrate these diverse educational approaches, facilitate communication between patients and clinicians in one-on-one or group forums, and afford better scalability to generate educational programs for a larger range of CKD patients.

We conducted a feasibility and preliminary efficacy study of the Modality Decision Program, a digital online educational program designed by specialty kidney care provider, Cricket Health, to prepare patients with advanced CKD for choosing a plan of care in the event that they reach ESRD [13]. We hypothesized that the program would have high usability and feasibility as well as high rates of program engagement. We also hypothesized that patients would have increased knowledge, self-efficacy, and confidence in choosing a treatment plan after completing the program and that the proportion of patients choosing in-home dialysis modalities would be higher after completion of the educational program.

## Methods

### Recruitment

Study participants were recruited in person, by mail, and by physician referral from the University of California, San Francisco (UCSF) Nephrology and Hypertension Clinic and the San Francisco Veterans Affairs Medical Center (SFVAMC) Renal Clinic. Patients at these clinics were first screened for eligibility using electronic health records and then mailed a letter explaining the study and providing contact information of the study coordinator. Screening of the medical record was primarily done manually by the study coordinator in the research team; additional efforts to enroll patients were made by enlisting the Clinical Research Services at UCSF to send out letters via their Recruitment Service. Two weeks after the recruitment letter was sent, the study coordinator called to inquire if they would like to join the study and to further evaluate eligibility. Participants were offered US $300 for completing the 2-month study. Flyers were also posted at UCSF and SFVAMC for eligible patients to contact the study team.

The study coordinator screened medical records, and the principal investigator confirmed the patient’s eligibility for the study based on the inclusion and exclusion criteria. Eligibility criteria included an estimated glomerular filtration rate (eGFR) <30 ml/min/1.73 m^2^ in the 6 months before enrollment, attendance at the renal clinic at least twice within the 18 months before enrollment, and documented discussion between patient and nephrologist about the potential need for dialysis in the future. Any 1 of the following resulted in exclusion: currently on dialysis, eGFR ≥10 ml/min/1.73 m^2^ over the past 6 months, age greater than 90 years, homelessness, inability to speak English, lack of phone or computer or internet access, lack of email access, dementia, severe cognitive impairment, blindness, deafness, or more than 2 hospitalizations during the last 6 months. All participants gave written informed consent. All procedures were in accordance with the Declaration of Helsinki (World Medical Association Declaration of Helsinki, 2000). The study was approved by the UCSF Human Research Protection Program Institutional Review Board (IRB study #16-19626), including waivers of written consent and Health Insurance Portability and Accountability Act authorization, and was registered on ClinicalTrials.gov (NCT02976220).

### The Modality Decision Program

The main objectives of the educational program were to (1) increase patient knowledge of ESRD treatment options, (2) help patients to prioritize options based on their lifestyle and values, and (3) build patients’ confidence in their treatment choice. The program design was informed by prior successful education programs [8]. The program was supplied as a responsive website accessible through a smartphone, tablet, or computer. Online digital content included 9 videos, consisting of one-on-one interviews with patients with ESRD; each patient describes how ESRD has affected his or her life and family and what factors influenced his or her choice of care for ESRD. The digital content also included answers to 129 frequently asked questions (FAQs), direct messaging, and individualized advice via online video from the study nurse, peer mentors (patients with ESRD who could share their experiences about treatment options), and a moderated patient discussion group. Samples of FAQs, nurse chat, and discussion board are shown in Multimedia Appendix 1.

Following the intake visit, participants were asked to engage with the educational program over a period of 4 weeks. During the 4 weeks, the study nurse followed up with the patient via messaging on the website and the participant had the opportunity to read educational materials and interact with the nurse and peer mentor group. For a participant to be considered adequately engaged with the program, the participant had to send at least one message to the nurse, mentors, or discussion board and also view at least one video or FAQ. These metrics of engagement were monitored by Cricket Health, and if a patient’s engagement was less than adequate on the online study platform during the 2 weeks following the intake visit, the study coordinator reminded the patient to engage with the platform. Program completion was marked by the end of the 4-week period or by the achievement of adequate engagement when not achieved by the end of the 4-week period. Upon completion of the educational program, participants discussed their preferred treatment option with the study nurse and addressed any remaining questions or open topics. On the basis of these discussions, the study nurse compiled an insights report summarizing the education the patient had received and any major concerns the patient expressed about their plan of care. After completion of the program, an insights report was sent to the patient and, if requested, their nephrologist.

### Study Visits

Each participant attended 3 study visits over a period of 2 months at the UCSF Nephrology and Hypertension Clinic. At the first study visit, the study coordinator conducted intake interviews, administered baseline surveys, took a medical history, verified eligibility, and obtained written informed consent. Participants were informed that completion of the study would require 8-14 hours over 2 months (2 hours for visit 1, 4-10 hours of engagement with the online program over 1 month, 1 hour for visit 2, and 1 hour for visit 3). The coordinator helped the participants log on to the website and gave them a tour of the program. The study nurse was present via online video during the intake visit. After participants completed the 4-week education program, they returned for a second study visit during which they repeated the survey administered at baseline. Participants attended a third study visit 1 month after completion of the intervention and were administered the same survey to evaluate whether the educational program had a durable effect. The third study visit occurred 1 month after the second (2 months after intake). During both the second and the third visits, participants were given the opportunity to provide qualitative feedback on the program.

### Survey Instruments

The study survey was developed specifically for this pilot study according to the educational content of the instructional materials. Cricket Health gave the research team access to the online subject matter so that we could ensure our surveys covered topics included in the educational program. We conducted a literature review of relevant surveys for patients with CKD to assess the content, vocabulary, and complexity of such surveys. CKD knowledge was assessed by 18 multiple-choice questions about knowledge of dialysis and treatment options modeled on those found in the Kidney Knowledge Survey [14], the ESRD Questionnaire [15], and the Chronic Hemodialysis Knowledge Survey [16]. Confidence in treatment choice was assessed by the Likert scale with the statement “I feel ready to choose a treatment option that would be best for me if I experience kidney failure.” CKD self-efficacy was assessed by 5 Likert items, including 1 from the CKD-Self Efficacy Survey (“I can actively share my experience of managing CKD with other patients”) [17] and 4 centered on self-care dialysis. A final question assessed the patient’s preferred treatment option should they reach ESRD based on a ranking of 5 choices. The survey also included 5 questions assessing patient satisfaction with the program. The complete patient survey is shown in Multimedia Appendix 2.

We contacted each patient’s nephrologist to inform them of the patient’s participation in the study and obtained consent from each nephrologist to contact him or her after the patient completed the study. To evaluate provider perspectives on the usefulness of the educational program for their patients’ individual care, we surveyed study participants’ nephrologists after each patient completed the final visit. The survey included 5 questions assessing whether the program helped the patient and the physician prepare for ESRD care (Multimedia Appendix 3).

### Statistical Design and Analysis

Initial analyses described the multistep enrollment into the study. For participants who completed the study, we describe engagement with the 4-week educational intervention program and demographic and clinical characteristics at baseline. In this single-group, pretest-posttest study design, patients served as their own control. On the basis of the results of a previous pretest-posttest study of a self-management education program among patients with CKD that evaluated changes in self-efficacy using a survey totaling 250 points [18], we calculated that a minimum sample size of 26 patients was needed to detect a change of 15 points on such a survey using a 2-tailed test and alpha level of .05.

We designed this study to include 2 posttest surveys to allow the primary evaluation of program effects immediately following program completion as well as a secondary evaluation of the durability of the effects of the educational intervention 4 weeks after program completion. Each participant’s CKD knowledge was scored as the percentage of answers correct out of the 18 CKD knowledge questions from the survey. Confidence in treatment choice and CKD self-efficacy were measured by participants’ responses on individual items according to a 5-point Likert scale. Additionally, scores from all 5 CKD self-efficacy questions were averaged to calculate an overall CKD self-efficacy score. In the main analyses, differences in scores on the survey completed before participating in the online program and after 4 weeks of participating in the program were evaluated by the Wilcoxon signed-rank test. For each measure, we also calculated the number and percentage of patients who improved their score. We described the number and percentage of patients choosing a specific preferred treatment option pre- and postintervention and used McNemar exact test to evaluate differences.

## Results

### Study Enrollment

**Figure 1:**
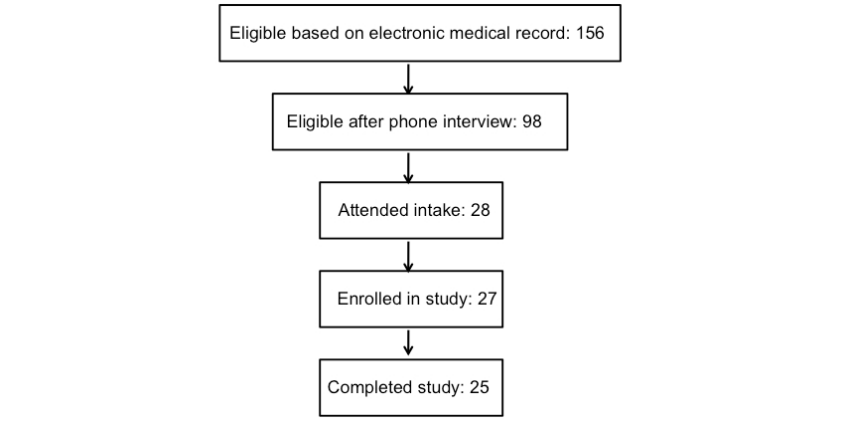
Recruitment and enrollment. A total of 2617 patients who had been seen at the University of California, San Francisco and San Francisco Veterans Affairs Medical Center renal clinics were either sent letters to notify them of the study or screened via the electronic medical record for eligibility. A total of 156 patients were potentially eligible based on initial screening; of these, we found 98 of those that responded to be eligible after the phone interview. Moreover, 27 of these enrolled into the study and 25 completed the study.

Overall, out of 2617 patients screened for eligibility, 156 were potentially eligible after screening the medical chart. After contacting these 156 patients, 98 were deemed eligible after the phone interview. Among 98 eligible patients, 48 were interested in the study and 39 scheduled an intake visit. Of these, 28 attended an intake visit and 25 completed the study (Figure 1).

### Baseline Characteristics

Characteristics of the 25 study participants who completed the study are summarized in Table 1. The mean age of participants was 65 years, 13 (13/25, 52%) were of non-white race, 17 (17/25, 68%) were male, and 13 (13/25, 52%) were retired. The average annual income among participants was US $45,000, and 9 (9/25, 36%) had college degrees. Moreover, 7 (7/25, 28%) had received dialysis in the past and 12 (12/25, 48%) reported having received education on ESRD treatment options before the study. A total of 20 (20/25, 80%) patients had been in the care of a nephrologist for over a year.

### Program Engagement

According to metrics collected by Cricket Health, each patient engaged with the program for a median of 5 (interquartile range: 2-6) hours over a mean of 32 (SD 14) days. Most participants used their own devices, although 3 participants (3/25, 12%) accessed the program using a shared or public computer. All participants viewed at least one FAQ, 24 (24/25, 96%) watched at least one video, and 21 (21/25, 88%) chatted with a nurse. Four participants needed more than 4 weeks to demonstrate adequate engagement with the online program and make a modality decision on the month 1 survey. Further details about patient engagement with the videos, FAQs, and discussion are summarized in Table 2.

### Preferred Treatment Option

At intake to the study, 32% (8/25) of the participants were unable to make a choice in the treatment plan; after completing the program, 100% (25/25) of the participants made a choice. The most commonly preferred treatment choice was kidney transplant, and the proportion of patients choosing this option increased from 12 (12/25, 48%) at intake to 21 (21/25, 84%) after program completion (*P*=.01). In a post hoc analysis, we determined patients’ preferred treatment choice in the case that a kidney transplant was not available or feasible (eg, with that answer option excluded). At intake, 4 (4/25, 16%) participants chose peritoneal dialysis if transplant was not available, compared with 13 (13/25, 52%) participants after program completion (*P*=.004). At intake, 2 (2/25, 8%) participants chose in-home hemodialysis if transplant was not available, compared with 4 (4/25, 16%) participants after program completion (*P*=.50). These proportions remained similar after program completion and 4 weeks later at the month 2 survey (Tables 3 and 4).

### Knowledge, Confidence, and Self-Efficacy

Mean scores for CKD knowledge and confidence in treatment choice improved significantly between surveys at intake of each study participant and program completion (*P* values<.03). The mean (SD) of all individual self-efficacy scores also increased significantly after the intervention, from 3.2 (1.4) to 4.1 (1.0; *P*<.001). Self-efficacy scores on 3 of the 5 individual Likert items improved, specifically: “My treatment would be just as good if I was responsible for my dialysis,” “I understand self-care dialysis,” and “I understand in-center dialysis” (*P* values ≤.005). In contrast, there was no statistically significant difference between intake and postintervention scores on the following statements: “I can actively share my experience of managing CKD with other patients” and “I could learn how to do self-care dialysis” (*P* values≥.06). Furthermore, most patients improved their scores between surveys performed at intake for each participant and program completion: 19 (19/25, 76%) on CKD knowledge, 15 (15/25, 60%) on confidence in treatment choice, and 20 (20/25, 80%) on overall CKD self-efficacy. Month 2 scores on assessments of CKD knowledge, confidence in treatment choice, and self-efficacy were similar to those from immediately after program completion (Table 5).

**Table 1:** Baseline characteristics of participants (N=25).

Characteristic		Statistics^a^
Age (years), mean (SD)		65 (15)
Female, n (%)		8 (32)
Latino, n (%)		2 (8)
**Race, n (%)**		
	Black	5 (20)
	White	12 (48)
	Other	8 (32)
**Employment status, n (%)**		
	Employed full- or part-time	6 (23)
	Retired	13 (52)
	Unemployed	3 (12)
Disability, n (%)		2 (8)
**Annual income US $, n (%)**		
	<25,000	9 (36)
	25,000-49,999	4 (16)
	50,000-99,999	4 (16)
	>100,000	6 (24)
**Highest level of education, n (%)**		
	High school diploma or general education diploma	4 (16)
	Associate’s degree	1 (4)
	Bachelor’s degree	9 (36)
	Master’s degree	4 (16)
	Doctorate	3 (12)
Uses the internet on shared or public computer, n (%)		3 (12)
Diabetes, n (%)		17 (68)
Prior or current tobacco use, n (%)		14 (56)
Time since CKD^b^ diagnosis (years), median (IQR^c^)		10 (4-23)
Estimated glomerular filtration rate (ml/min/1.73 m^2^), mean (SD)		21 (6)
**CKD cause, n (%)**		
	Glomerulonephritis	5 (20)
	Diabetes	4 (16)
	Hypertension	3 (12)
	Medication adverse effect	2 (8)
	Vasculitis	1 (4)
	Polycystic kidney disease	1 (4)
	Unknown	9 (36)
**History of dialysis, n (%)**		
	Peritoneal dialysis	1 (4)
	Hemodialysis	6 (24)
Vascular access, n (%)		3 (12)
Evaluated for transplant, n (%)		13 (52)
Active on transplant list, n (%)		7 (28)
**“How long have you been seeing your current nephrologist?” n (%)**		
	5-8 months	2 (8)
	9-12 months	3 (12)
	>12 months	20 (80)
**“I have received education about treatment options for chronic kidney disease prior to participating in this study,” n (%)**		
	Agree or strongly agree	12 (48)
	Neutral or unsure	7 (28)
	Disagree or strongly disagree	6 (24)

^a^Categorical data are shown as n (%); continuous data are summarized as mean (SD) or median (IQR). All data were collected via self-report.

^b^CKD: chronic kidney disease.

^c^IQR: interquartile range.

**Table 2:** Participation and engagement during the 4-week educational program (N=25).

Measure		Statistics^a^
**General**		
	Days required to complete program, mean (SD)	32 (14)
	Hours active on program, median (IQR^b^)	5 (2-6)
**Videos**		
	Viewed at least one video, n (%)	24 (96)
	Number of unique videos viewed (out of 9), median (IQR)	8 (5-9)
	Total number of video views), median (IQR)	10 (8-21)
**Frequently asked questions**		
	Viewed at least one frequently asked question, n (%)	25 (100)
	Number of unique frequently asked questions viewed (out of 129), median (IQR)	24 (10-98)
	Total number of frequently asked question views, median (IQR)	32 (15-118)
**Chat communication**		
	Number of messages sent to nurse, mentors, or discussion board, median (IQR)	11 (4-19)
	Number of conversations viewed on discussion board, median (IQR)	27 (13-47)
	Chatted with nurse, n (%)	21 (84)
	Chatted with mentor, n (%)	6 (24)
	Chatted with group, n (%)	11 (44)

^a^Categorical data are shown as n (%); continuous data are summarized as mean (SD) or median (IQR). Data were collected by Cricket Health.

^b^IQR: interquartile range.

**Table 3:** Preferred treatment modality choice, if transplant is a feasible choice.

Treatment choice		n (%)^a^
**Intake survey (N=25)**		
	Kidney transplant	12 (48)
	Conservative management	2 (8)
	Peritoneal dialysis	1 (4)
	Home hemodialysis	1 (4)
	In-center hemodialysis	1 (4)
	None/unsure	8 (32)
**Month 1 survey (N=25)**		
	Kidney transplant	21 (84)^b^
	Conservative management	2 (8)
	Peritoneal dialysis	1 (4)
	Home hemodialysis	1 (4)
	In-center hemodialysis	0 (0)
	None/unsure	0 (0)
**Month 2 survey (N=24)**		
	Kidney transplant	19 (79)
	Conservative management	2 (8)
	Peritoneal dialysis	1 (4)
	Home hemodialysis	2 (8)
	In-center hemodialysis	0 (0)
	None/unsure	0 (0)

^a^Categorical data are shown as n (%). Data obtained from surveys conducted at intake of each study participant, after 4 weeks of engagement and completion of the Modality Decision Program, and then 4 weeks after completing the Modality Decision Program.

^b^*P* value for tested differences in proportions between initial and month 1 survey, using McNemar exact test was .01.

**Table 4:** Preferred treatment modality choice, if transplant is not a feasible choice.

Treatment choice with transplant option removed		n (%)^a^
**Intake survey (N=25)**		
	*[removed: Kidney transplant]* ^b^	—^c^
	Conservative management	6 (24)
	Peritoneal dialysis	4 (16)
	Home hemodialysis	2 (8)
	In-center hemodialysis	5 (20)
	None/unsure	8 (32)
**Month 1 survey (N=25)**		
	*[removed: Kidney transplant]*	—
	Conservative management	6 (24)
	Peritoneal dialysis	13 (52)^d^
	Home hemodialysis	4 (16)^e^
	In-center hemodialysis	2 (8)
	None/unsure	0 (0)
**Month 2 survey (N=24)**		
	*[removed: Kidney transplant]*	—
	Conservative management	3 (13)
	Peritoneal dialysis	12 (50)
	Home hemodialysis	6 (25)
	In-center hemodialysis	3 (13)
	None/unsure	0 (0)

^a^Categorical data are shown as n (%). Data obtained from surveys conducted at intake of each study participant, after 4 weeks of engagement and completion of the Modality Decision Program, and then 4 weeks after completing the Modality Decision Program.

^b^In a post hoc analysis, we determined patients’ preferred treatment choice in the case that a kidney transplant was not available or feasible (eg, with that answer option excluded).

^c^Not applicable.

^d^*P* value for tested differences in proportions between initial and month 1 survey, using McNemar exact test was .004.

^e^*P* value for tested differences in proportions between initial and month 1 survey, using McNemar exact test was .50.

**Table 5:** Chronic kidney disease knowledge, confidence, and self-efficacy.

Measure			Statistics^a^	*P* value^b^
**CKD^c^ knowledge (% correct from 18 CKD knowledge questions)**				<.001
	Initial survey, mean (SD)		65 (56)	
	Month 1 survey (main effect), mean (SD)		83 (14)	
	Month 2 survey (durability), mean (SD)		86 (11)	
	Patients who improved after program, n (%)		19 (76)	
**Confidence in treatment choice (Likert scale 1-5): “I feel ready to choose a treatment option that would be best for me if I experience kidney failure”**				.03
	Initial survey, mean (SD)		3.2 (1.4)	
	Month 1 survey, mean (SD)		4.1 (1.0)	
	Month 2 survey, mean (SD)		4.3 (0.7)	
	Patients who improved after program, n (%)		15 (60)	
**CKD self-efficacy (Likert scale 1-5)**				
	**Average CKD self-efficacy score**			<.001
		Initial survey, mean (SD)	3.7 (0.7)	
		Month 1 survey, mean (SD)	4.3 (0.5)	
		Month 2 survey, mean (SD)	4.3 (0.6)	
		Patients who improved after program, n (%)	20 (80)	
	**“I can actively share my experience of managing CKD with other patients.”^d^**			.12
		Initial survey, mean (SD)	4.1 (0.6)	
		Month 1 survey, mean (SD)	4.2 (0.6)	
		Month 2 survey, mean (SD)	4.1 (0.9)	
		Patients who improved after program, n (%)	5 (20)	
	**“My treatment would be just as good if I was responsible for my dialysis.”**			.002
		Initial survey, mean (SD)	3.6 (0.8)	
		Month 1 survey, mean (SD)	4.3 (0.6)	
		Month 2 survey, mean (SD)	4.3 (0.6)	
		Patients who improved after program, n (%)	14 (56)	
	**“I could learn how to do self-care dialysis.”**			.06
		Initial survey, mean (SD)	4.2 (0.6)	
		Month 1 survey, mean (SD)	4.4 (0.7)	
		Month 2 survey, mean (SD)	4.5 (0.5)	
		Patients who improved after program, n (%)	6 (24)	
	**“I understand self-care dialysis.”**			<.001
		Initial survey, mean (SD)	3.1 (1.2)	
		Month 1 survey, mean (SD)	4.2 (0.8)	
		Month 2 survey, mean (SD)	4.2 (0.6)	
		Patients who improved after program, n (%)	16 (64)	
	**“I understand in-center dialysis.”**			.005
		Initial survey, mean (SD)	3.4 (1.3)	
		Month 1 survey, mean (SD)	4.2 (0.6)	
		Month 2 survey, mean (SD)	4.2 (0.7)	
		Patients who improved after program, n (%)	12 (48)	

^a^Categorical data are shown as n (%); continuous data (scores) are summarized as mean (SD). Data obtained from surveys.

^b^*P* value calculated by Wilcoxon signed-rank test for the difference between initial values and values at month 1 study visit.

^c^CKD: chronic kidney disease.

^d^Self-efficacy statement drawn from CKD Self-Efficacy instrument developed by Lin et al [17].

### Patient and Physician Satisfaction

Overall, both patients and physicians were satisfied with the educational program. All patients responded with a score of ≥4 (out of 5) that they would recommend the program to a friend or family member, that the program was valuable in making a treatment choice, and that the website was easy to use. Among 22 nephrologists of program participants, 21 (21/25, 95%) indicated that the program helped their patients prepare for ESRD care, 18 (18/25, 82%) indicated that the program helped the patient choose a care plan for ESRD, and 21 (21/25, 95%) indicated that the program helped the physician learn about the patient’s lifestyle and care preferences and also made it easier for the physician to care for the patient. Only 1 (1/25, 5%) indicated that the program could be improved by including more information on management.

## Discussion

In this study, we found that delivering digitally enabled ESRD education to patients in clinical care is feasible using the Modality Decision Program developed by Cricket Health. Patients demonstrated a high level of engagement, and they were able to complete the program and make a choice in the plan of ESRD care. Kidney transplant was the most common first choice for ESRD care. Among choices for dialysis modality, we observed that patients were more likely to choose self-care, in-home dialysis options (particularly peritoneal dialysis) after completing the education program. The Cricket Health program increased patients’ knowledge of ESRD treatment options as well as their self-efficacy regarding self-care, in-home dialysis modalities.

The importance of predialysis patient education is well established [19]. Prior research has shown that educational interventions in predialysis patients may delay the need for dialysis [20], encourage patients to agree to early vascular access for hemodialysis [21], and even have long-term survival benefits [22]. Manns et al conducted a randomized controlled study of an educational program that included written materials, a video, and small group sessions and found that it improved awareness and preference for in-home, self-care dialysis [8]; additional programs centered around in-person education have been shown to increase the likelihood of patients choosing peritoneal dialysis rather than hemodialysis [9-11]. Such in-person programs are labor- and personnel-intensive and may not easily be scaled up to reach a larger number of patients. Online media is a rapidly expanding topic of research for self-management of patients with a number of chronic diseases, including CKD [23-25]. Several internet-based self-management programs have been developed for patients with ESRD [26-28]. Few internet-based educational programs for CKD have been tested for the specific purpose of educating patients with predialysis CKD on options of care for ESRD.

The Modality Decision Program developed by Cricket Health builds on these prior educational programs and also addresses the need for education that can be digitally distributed to a wider audience of CKD patients. The online, digital format increases accessibility, addresses individual lifestyles and preferences, and allows the patient to learn about modality options at his or her own pace. Throughout the educational materials, patients are encouraged to choose a treatment option that fits into his or her own lifestyle. This individualized approach likely contributes to the success we observed in improving patients’ knowledge of ESRD, willingness to choose peritoneal dialysis when transplant is not an option, and to enhance patients’ confidence and self-efficacy regarding treatment choice.

In addition to the quantifiable effects on patient knowledge and treatment choice, the Modality Decision Program may have an emotional impact. The educational program incorporates emotional support for patients by integrating peer mentors who already have ESRD and facilitating one-on-one communication with the study nurse. During the program, these sources of emotional support are available through email and chat, which is likely to be more convenient and accessible than conventional in-person support groups. As other researchers have noted, a patient’s effort to learn about CKD can be undermined by uncertainty and fear [14]. Although most patients’ confidence and self-efficacy improved after the program, multiple patients commented on an emotional response to it, and 1 study participant interrupted his participation in the program because of the emotional burden imposed by learning about CKD. Prior researchers have recognized the therapeutic potential of patient-centered educational programs that emphasize support and community, discuss the emotional aspects of CKD, and teach coping skills [29]. Subsequent programs developed by Cricket Health aim to give patients additional emotional support as they learn about CKD and ESRD. For example, the current Wellness Management Program developed by Cricket Health integrates a social worker into the educational team for further emotional support.

Patients with kidney disease often have other comorbidities that complicate their plan of care. Educational programs should take into account the complex medical condition of many CKD patients and provide educational content addressing the intersections of comorbid diseases, such as diabetes and CKD. Subsequent programs developed by Cricket Health have focused on disease-specific aspects of CKD management, including blood pressure management, congestive heart failure, diet, and nutrition.

Enrolling patients with CKD into clinical trials is challenging. Reasons for low enrollment of CKD patients into clinical trials may include their high comorbidity burden, necessitating frequent doctor visits or hospitalizations that make the patient unable to attend study visits, and their high rates of disability [30]. We observed that out of over 2000 screened patients, 98 were eligible for the study after the phone interview. About 50% (49/98) of eligible patients were interested and about 50% (49/98) of these interested participants enrolled in and completed the study. This low response and participation rate is consistent with prior literature; taking this into consideration, if further studies are planned to evaluate this or other educational programs for patients with advanced CKD, they would likely have to be multicenter to enroll more patients.

This study has several limitations. The patient survey we developed to evaluate changes in patient knowledge and self-efficacy used items from validated surveys but was not validated in our population. The study design involved a single group of patients, and the results might be less conclusive than if it were structured as a randomized controlled trial. Specifically, it is possible that the observed improvement may have occurred for reasons unrelated to the program, although there are no other known potential sources of improvement. Our geographical focus in the San Francisco Bay Area and the requirement that participants be screened for eligibility by phone may have selected a higher proportion of affluent, tech-savvy participants that might not be representative of the broader US population. However, our sample of participants was racially diverse and represented a relatively broad socioeconomic spectrum. One of our inclusion criteria was that the patient must have had a documented discussion of dialysis with his or her nephrologist during a clinic visit. We felt it was important for ethical reasons to make sure the patient was not informed for the first time by the study team about the possible need of dialysis in the future; however, these criteria did limit the number of patients in the study and could result in a more informed sample with greater knowledge of ESRD treatment options than the general US population of patients with advanced CKD. We also felt that excluding patients with more than 2 hospitalizations in the past year or no internet access would help expedite timely completion of the study, but these criteria did also limit generalizability as well as our sample size.

In conclusion, we have conducted a pretest-posttest study among 25 patients with advanced CKD to evaluate the potential impact of the Modality Decision Program developed by Cricket Health for increasing patients’ CKD knowledge, self-efficacy, ability to make a choice in ESRD treatment modality, and preference for in-home therapies. After completing the Modality Decision Program, patients had improved knowledge, confidence, and self-efficacy; were able to make a choice of treatment modality for ESRD; and were more likely to choose self-care, in-home dialysis therapies as their preferred dialysis modality. In summary, implementation of a digital ESRD education program is feasible and may be effective in facilitating patients’ decisions about renal replacement therapies. Larger studies are necessary to understand whether the program affects clinical outcomes.

## Appendix

Multimedia Appendix 1 Example of the online digital content of the educational program.

Multimedia Appendix 2 Patient survey.

Multimedia Appendix 3 Provider survey.

## Abbreviations

CKD: chronic kidney disease
eGFR: estimated glomerular filtration rate
ESRD: end-stage renal disease
FAQ: frequently asked question
IQR: interquartile range
SFVAMC: San Francisco Veterans Affairs Medical Center
UCSF: University of California, San Francisco
