# Supplementary material for: A Digital Modality Decision Program for Patients With Advanced Chronic Kidney Disease
Source: JMIR Form Res. 2019 Feb 6;3(1):e12528. doi: 10.2196/12528 (PMC6381409; doi:10.2196/12528)
Supplement: Multimedia Appendix 1 [file formative_v3i1e12528_app1.pdf]

Supplement 1: Online Materials

Examples of the Cricket Health Platform on a Laptop and Phone

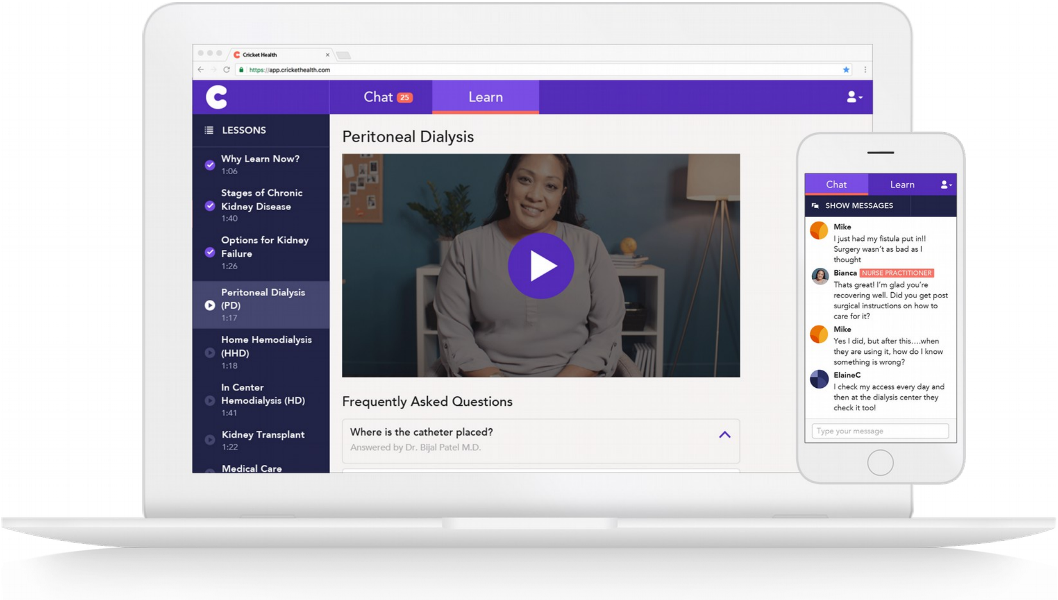

An Example of a Frequently Asked Question (FAQ)

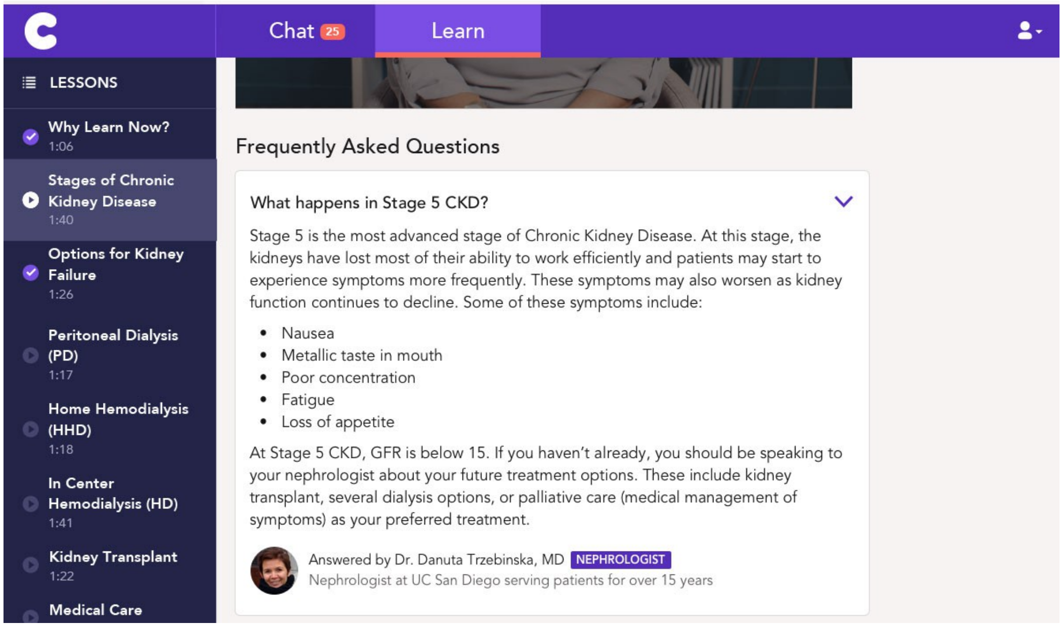

An Example of a Nurse Chat

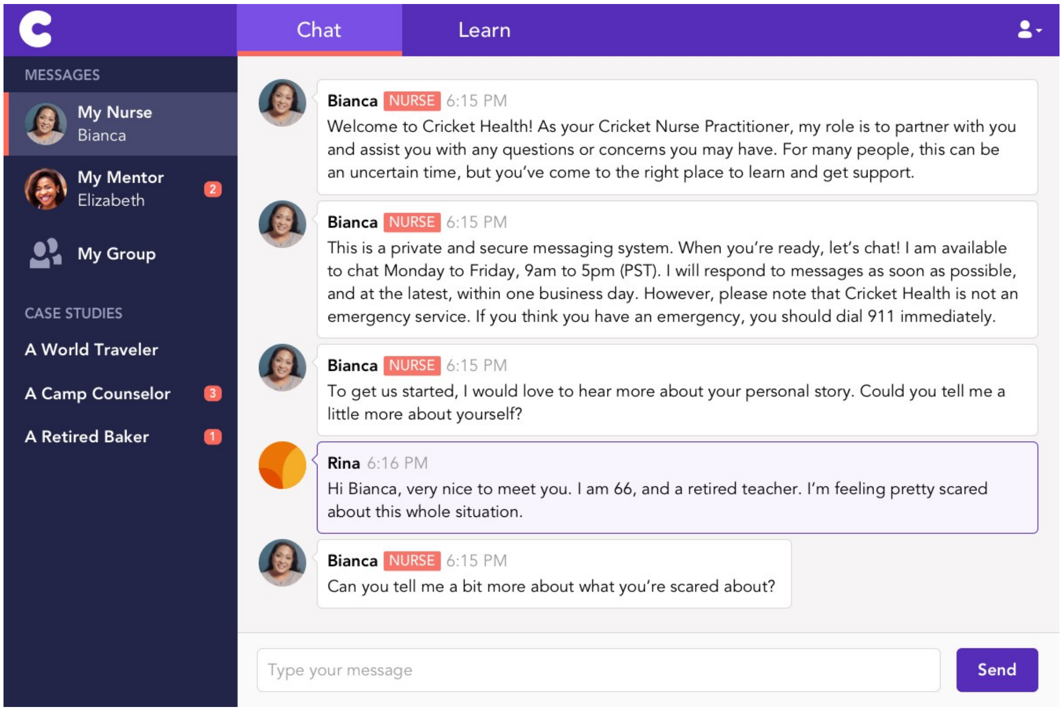

An Example of a Mentor Chat

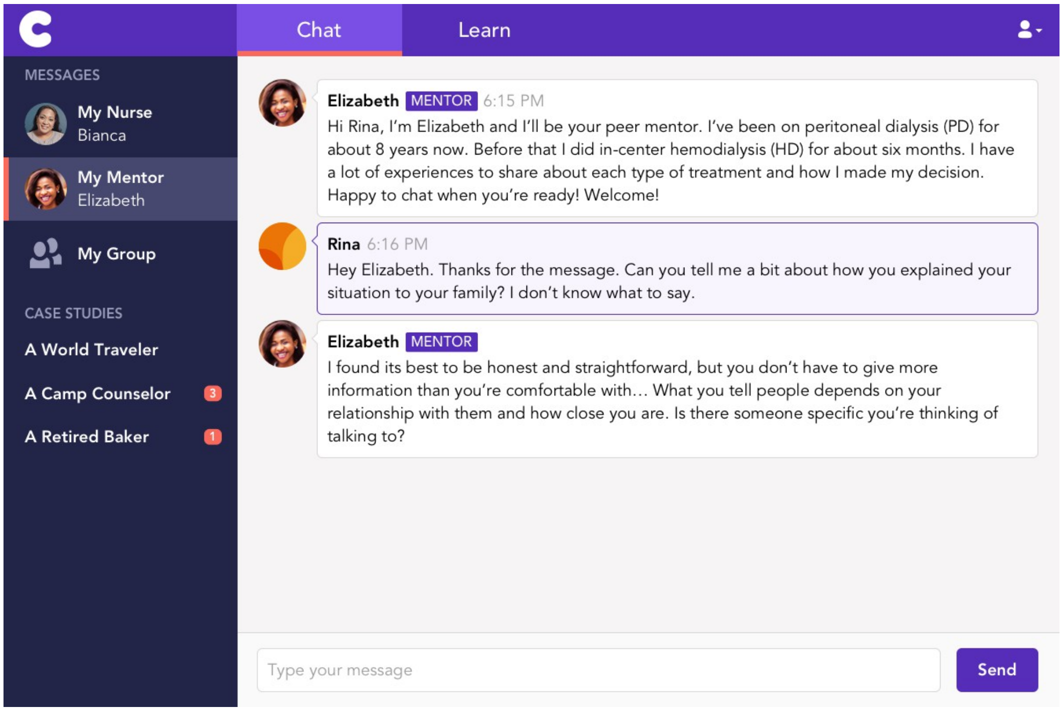

An Example of a Discussion Board

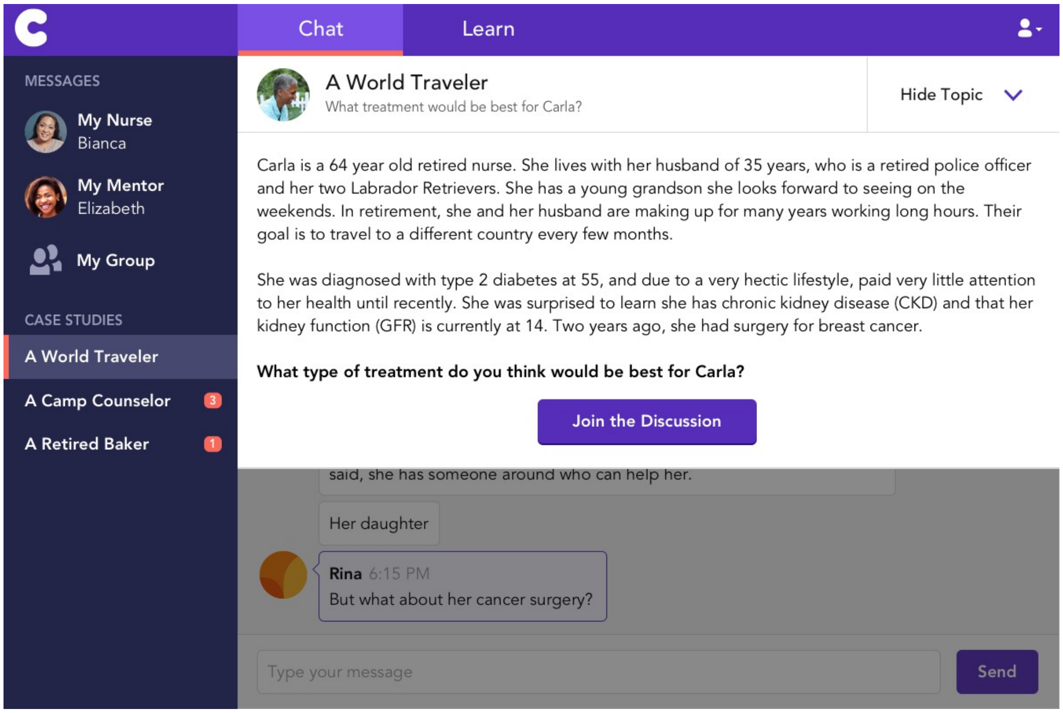

Note: Text and messages above are for illustrative purposes only.

Table of Contents

Topics Covered:

1. Why Learn Now?
2. Stages of Chronic Kidney Disease
3. Options for Kidney Failure
4. Peritoneal Dialysis
5. Home Hemodialysis
6. In Center Hemodialysis
7. Kidney Transplant
8. Medical Care Without Dialysis
9. Making a Decision

1. Why Learn Now?

Video Length: 2 minutes, 37 seconds

Examples of Frequently Asked Questions (FAQs) covered:

- If I have CKD, why don't I feel anything yet?
- Is there a cure for CKD? How can I make it go away?
- I'm feeling really scared and overwhelmed about my CKD diagnosis. What do I do?

2. **Stages of Chronic Kidney Disease**

Video Length: 2 minutes, 21 seconds

Examples of Frequently Asked Questions (FAQs) covered:
- What are the GFR Values for each stage of CKD?
- What happens in Stage 4 CKD?
- What happens in Stage 5 CKD?

3. **Options for Kidney Failure**

Video Length: 2 minutes, 39 seconds

Examples of Frequently Asked Questions (FAQs) covered:
- If I get kidney failure, what do I do about it? What are my options?
- Can I decide not to get kidney replacement therapy if I have kidney failure?
- If I choose dialysis, when would I have to start?

4. **Peritoneal Dialysis**

Video Length: 2 minutes, 49 seconds

Examples of Frequently Asked Questions (FAQs) covered:
- What do you like and dislike about PD?
- What does PD feel like?
- How do I get the catheter? When will I need it?

5. **Home Hemodialysis**

Video Length: 2 minutes, 42 seconds

Examples of Frequently Asked Questions (FAQs) covered:
- What are pros and cons of home hemodialysis?
- What does HHD feel like?
- How does the blood get to the machine? What is vascular access?

6. **In Center Hemodialysis**

Video Length: 2 minutes, 1 second

Examples of Frequently Asked Questions (FAQs) covered:
- What are the pros and cons of HD?
- What's the difference between Home Hemodialysis (HHD) and In Center Hemodialysis (HD)?

- How often will I need to go into the clinic to get HD treatments?

7. Kidney Transplant

Video Length: 2 minutes, 44 seconds

Examples of Frequently Asked Questions (FAQs) covered:

- How do I know if I am a good candidate for transplant?
- Once I get a new kidney, am I cured? What if my new kidney stops working?
- What difference does it make if I get a living donor vs a deceased donor?

8. Medical Care Without Dialysis

Video Length: 2 minutes, 47 seconds

Examples of Frequently Asked Questions (FAQs) covered:

- How long will I live if have very low kidney function and I don’t get treatment?
- What is the difference between hospice and palliative care?
- Can I manage kidney failure with diet alone or natural therapies?

9. Making a Decision

Video Length: 1 minute, 32 seconds

Examples of Frequently Asked Questions (FAQs) covered:

- What did you think about when you chose your treatment for kidney failure?
- Who makes the final decision about my treatment?
- How far in advance would I need to prepare for dialysis? What would I need to do?
