# Supplementary material for: A Digital Modality Decision Program for Patients With Advanced Chronic Kidney Disease
Source: JMIR Form Res. 2019 Feb 6;3(1):e12528. doi: 10.2196/12528 (PMC6381409; doi:10.2196/12528)
Supplement: Multimedia Appendix 2 [file formative_v3i1e12528_app2.pdf]

POST-STUDY SURVEY

Health Options Patient Educations (Hope)
UCSF Study

1. What is GFR?

A. Glomerular filtration rate. A number from a blood test that tells approximately how much kidney function is left

B. Good flow rate. A number that tells how fast someone can empty their bladder of urine

C. Gain for real. A number that tells how someone's kidney function is improving

D. Glucose function rate. A measurement of sugar in the blood

E. I don't know

2. What is dialysis?

A. Dialysis is a treatment to heal kidneys and bring their function back to normal

B. Dialysis is a treatment to prevent kidneys from failing

C. Dialysis is a treatment to clean the body of toxins and excess fluid

D. Dialysis is a device that is placed inside the body when someone has kidney failure

E. I don't know

3. Severe CKD (Chronic Kidney Disease) stage 4 or 5 can be cured by:

A. It is not curable

B. Special kidney diet

C. Exercise

D. Drinking a lot of water

E. I don't know

4. What are possible symptoms of CKD (Chronic Kidney Disease)?

A. Shortness of breath

B. No symptoms at all

C. Nausea and/or vomiting

D. Increased fatigue

E. All of the above

5. You have to be a dialysis patient before receiving a kidney transplant

A. True

B. False

C. I don't know

6. The kidney used in a transplant can come from:

A. A relative (close or distant family member)

B. A non-relative (for example: a friend, neighbor or coworker)

C. A deceased organ donor (a person who offered to donate a kidney after death)

D. Any of the above

E. I don't know

7. If a patient with kidney failure (End Stage Renal Disease) decides not to start dialysis, what are the treatment options?

A. None. All the doctor's appointments will be canceled

B. Treatment of symptoms with medications and support to keep the patient as comfortable as possible for as long as possible

C. Have the family or doctors convince the patient to change his or her mind to start dialysis

D. Go on a special diet that will heal the kidneys

E. I don't know

8. Which of the following is a form of in-home treatment that uses patient's abdomen (belly area) to remove toxins from the body?

A. Conservative management

B. Kidney transplant

C. Peritoneal dialysis (PD)

D. Home Hemodialysis (HHD)

E. In-Center Hemodialysis (IHD)

F. I don't know

9. Which of the following is a form of in-home treatment that uses a machine to filter blood outside of the body?

A. Conservative management

B. Kidney transplant

C. Peritoneal dialysis (PD)

D. Home Hemodialysis (HHD)

E. In-Center Hemodialysis (IHD)

F. I don't know

10. Which of the following is a form of treatment where healthcare professionals use a machine to filter blood in the clinic?

A. Conservative management
B. Kidney transplant
C. Peritoneal dialysis (PD)
D. Home Hemodialysis (HHD)
E. In-Center Hemodialysis (IHD)
F. I don't know

11. Which of the following is a form of medical treatment to keep the patient as comfortable as possible for as long as possible?

A. Conservative management
B. Kidney transplant
C. Peritoneal dialysis (PD)
D. Home Hemodialysis (HHD)
E. In-Center Hemodialysis (IHD)
F. I don't know

12. Which of the following is a form of treatment in which a patient undergoes a surgery to receive a donated kidney?

A. Conservative management
B. Kidney transplant
C. Peritoneal dialysis (PD)
D. Home Hemodialysis (HHD)
E. In-Center Hemodialysis (IHD)
F. I don't know

13. How often is In-Center Hemodialysis (IHD) typically done?

A. Only when the patient feels sick
B. Once a week
C. Three times a week
D. Every day
E. I don't know

14. How often is Peritoneal Dialysis (PD) typically done?

A. Only when the patient feels sick
B. Once a week
C. Three times a week
D. Every day
E. I don't know

15. Which type of dialysis does NOT require needles?

A. Peritoneal dialysis (PD)
B. Home Hemodialysis (HHD)
C. In-Center Hemodialysis (IHD)
D. I don't know

16. A patient who is trained to do self-care dialysis at home can be just as successful as a patient who gets dialysis from nurses at a clinic.

A. True
B. False
C. I don't know

17. Which of the following is NOT a benefit of home hemodialysis (HHD)?

A. Flexible treatment schedule
B. Less fluid and diet restrictions compared to in-center dialysis
C. Improved heart function and blood pressure control
D. No infections
E. I don't know

18. Which dialysis treatment options allow patients to travel?

A. Peritoneal dialysis (PD)
B. Home Hemodialysis (HHD)
C. In-Center Hemodialysis (IHD)
D. All of the above
E. None of the above, dialysis patients cannot travel

19. I have received education about treatment options for kidney failure (End Stage Renal Disease).

Strongly disagree	Disagree	Neutral or unsure	Agree	Strongly agree
1	2	3	4	5

20. I feel ready to choose a treatment option that would be best for me if I experience kidney failure (End Stage Renal Disease).

Strongly disagree	Disagree	Neutral or unsure	Agree	Strongly agree
1	2	3	4	5
If you select this option, skip to question 26	*If you select this option, skip to question 26*	*If you select this option, skip to question 26*		

If I experience kidney failure (end stage renal disease) then my order of preference of treatment options would be:

	Most prefered				Least Preferred
21. Kidney Transplant	1	2	3	4	5
22. Conservative Management	1	2	3	4	5
23. Peritoneal Dialysis (PD)	1	2	3	4	5
24. Home Hemodialysis (HHD)	1	2	3	4	5
25. In-Center Hemodialysis (IHD)	1	2	3	4	5

26. I can actively share my experience of managing CKD with other patients.

Strongly disagree	Disagree	Neutral or unsure	Agree	Strongly agree
1	2	3	4	5

27. My treatment would be just as good if I was responsible for my dialysis.

Strongly disagree	Disagree	Neutral or unsure	Agree	Strongly agree
1	2	3	4	5

28. I could learn how to do self-care dialysis.

Strongly disagree	Disagree	Neutral or unsure	Agree	Strongly agree
1	2	3	4	5

29. I understand self-care dialysis.

Strongly disagree	Disagree	Neutral or unsure	Agree	Strongly agree
1	2	3	4	5

30. I understand in-center dialysis.

Strongly disagree	Disagree	Neutral or unsure	Agree	Strongly agree
1	2	3	4	5

31. Based on your experience, how likely are you to recommend HOPE program to a friend or family member should they need it?

Not likely at all — Extremely likely

1 · · · 2 · · · 3 · · · 4 · · · 5 · · · 6 · · · 7 · · · 8 · · · 9

32. The HOPE program was valuable to me in making my treatment choice.

Strongly disagree	Disagree	Neutral or unsure	Agree	Strongly agree
1	2	3	4	5

33. I found the HOPE website easy to use.

Strongly disagree	Disagree	Neutral or unsure	Agree	Strongly agree
1	2	3	4	5

34. Choose 3 features of HOPE that you found most valuable.

A. Educational Videos

B. Frequently Asked Questions

C. Discussion with Patient Peers

D. Discussion with Mentors

E. Group Exercises

F. 1 on 1 Nurse Discussion

G. Treatment Preferences Report

35. What can we do to improve the HOPE program (optional?):
